# Diagnosis and Treatment Options in Pigmented Villonodular Synovitis of the Knee: A Narrative Review

**DOI:** 10.3390/jcm14165857

**Published:** 2025-08-19

**Authors:** Andrea De Fazio, Giovan Giuseppe Mazzella, Tommaso Greco, Chiara Comisi, Camillo Fulchignoni, Giulio Maccauro, Carlo Perisano

**Affiliations:** Orthopedics and Trauma Surgery Unit, Department of Ageing, Neurosciences, Head-Neck and Orthopedics Sciences, Fondazione Policlinico Universitario Agostino Gemelli IRCCS, 00168 Rome, Italy; andrea.defazio01@icatt.it (A.D.F.); ggmazzella@gmail.com (G.G.M.); tommaso.greco91@libero.it (T.G.); chiara.comisi01@icatt.it (C.C.); camillo.fulchignoni@policlinicogemelli.it (C.F.); giulio.maccauro@policlinicogemelli.it (G.M.)

**Keywords:** pigmented villonodular synovitis, tenosynovial giant cell tumor, knee joint, synovectomy, arthroscopic surgery

## Abstract

**Background**: Pigmented villonodular synovitis (PVNS), also known as tenosynovial giant cell tumor, is a rare proliferative disorder of the synovial membrane that primarily affects the knee joint. Despite advances in imaging and surgical techniques, diagnosis is often delayed, and optimal treatment remains debated. **Methods**: A Narrative review was conducted according to PRISMA guidelines using PubMed, MEDLINE, and Scopus databases from January 2000 to December 2024. Studies reporting on epidemiology, clinical features, imaging, treatment, and outcomes of PVNS were included. **Results**: Sixty-six studies encompassing 120 patients were included. The majority of cases were diffuse PVNS. MRI was the most effective imaging tool. Arthroscopic synovectomy was the most common treatment, though recurrence rates remained high, particularly in diffuse forms. Adjuvant treatments, including radiosynoviorthesis and biologic therapies such as infliximab or pexidartinib, were employed in recurrent or unresectable cases. **Conclusions**: Early diagnosis and complete surgical excision remain the mainstay of treatment. Combined open and arthroscopic approaches are recommended in diffuse PVNS. Further prospective studies are needed to define optimal long-term management.

## 1. Introduction

Pigmented villonodular synovitis (PVNS), also known as tenosynovial giant cell tumor (TGCT), is an uncommon but clinically significant proliferative disorder of the synovial membrane, tendon sheaths, and bursae. Originally described by Chassaignac in 1852 and subsequently characterized in detail by Jaffe et al. in 1941, PVNS is described as a spectrum of benign neoplastic conditions distinguished by hemosiderin-laden macrophages, multinucleated giant cells, lipid-laden histiocytes, and inflammatory infiltrates within a hyperplastic synovium [1,2].

### 1.1. Epidemiology

The incidence of PVNS is approximately 1.8 per million people per year, with the knee being the most frequently involved joint, accounting for up to 80% of cases, followed by the hip, ankle, and shoulder [3,4,5,6,7].

It affects adults between the third and fifth decades of life; however, several cases have also been reported in the pediatric population [8,9]. Although PVNS shows no consistent sex predilection in the general population, isolated reports have noted a slight female predominance among pediatric patients [8,10,11,12,13].

### 1.2. Clinical Presentation

PVNS is classified into two different clinical presentations: localized PVNS (LPVNS) and diffuse PVNS (DPVNS). The localized form is generally confined, often intra-articular, and accessible to arthroscopic excision with relatively low recurrence rates [14]. In contrast, DPVNS involves a more extensive and aggressive proliferation of synovial tissue, often with extra-articular extension, joint effusion, and significant erosive damage to adjacent cartilage and bone [15,16].

Clinically, PVNS presents with non-specific symptoms such as monoarticular pain, swelling, recurrent joint effusion, and limited range of motion. In some cases, mechanical symptoms such as locking and instability are observed, especially when the lesion is located near critical intra-articular structures such as the anterior cruciate ligament [14]. Due to its insidious onset and overlapping clinical features with other arthropathies, including rheumatoid arthritis, juvenile idiopathic arthritis, and synovial cysts, PVNS is often misdiagnosed or diagnosed late [8,17,18].

### 1.3. Diagnosis

Radiographic studies typically reveal nonspecific findings such as joint space narrowing or periarticular erosions. MRI remains the gold standard for diagnosis due to its sensitivity to hemosiderin deposition, which appears as low signal intensity on T1- and T2-weighted images, with characteristic “blooming” artifacts on gradient echo sequences [19]. Contrast-enhanced sequences have been shown to demonstrate the extent of synovial involvement and differentiate PVNS from other intra-articular lesions [14,20].

Histopathologically, PVNS shows villous and nodular proliferation of the synovium, accompanied by hemosiderin-laden macrophages, foamy histiocytes, and multinucleated giant cells. Recent molecular analyses have identified colony-stimulating factor 1 (CSF1) expression in neoplastic synovial cells, suggesting a translocation-induced paracrine growth mechanism that recruits non-neoplastic inflammatory cells expressing CSF1 receptors [21].

### 1.4. Treatment

Surgical excision of the affected synovium remains the gold standard of treatment. For LPVNS, arthroscopic synovectomy is generally considered sufficient and is associated with low morbidity and excellent functional outcomes [9]. In DPVNS, complete synovectomy is technically challenging and often requires a combination of open and arthroscopic approaches to achieve optimal results [15,16]. Although the implementation of rigorous resection protocols, recurrence rates persist at considerable levels, ranging from 18% to 46%, depending on the form and extent of excision [19,22].

Adjuvant treatment options are often considered in the management of recurrent or incompletely resectable PVNS. These include the following:

- Radiosynoviorthesis, using intra-articular injection of radioactive compounds (e.g., osmic acid, yttrium-90), has shown promising results [9].
- Biologic therapies, such as intra-articular infliximab in refractory cases, have shown symptomatic and histological improvements by downregulating TNF-α-mediated synovial proliferation [17].
- Experimental approaches, such as ozone therapy, may offer alternative options in selected cases of recurrence [22].

Moreover, the use of targeted small-molecule inhibitors like pexidartinib, a selective Colony-Stimulating Factor 1 inhibitor (CSF1R), has recently transformed therapeutic strategies. Clinical trials have demonstrated significant tumor regression and symptom relief in patients with unresectable or recurrent disease. However, these agents are not without adverse effects, and their long-term safety profiles remain under evaluation [21].

It is important to consider how PVNS manifests in different clinical contexts.

Cases of multifocal disease involving both intra- and extra-articular structures may present a diagnostic and therapeutic dilemma [23]. In addition, it has been observed that associations with systemic conditions, such as rheumatoid arthritis, can add further complexity to the clinical presentations and potentially lead to delays in diagnosis [18].

Unusual presentations, such as PVNS following total knee arthroplasty, challenge conventional postoperative complication patterns [16]. Pediatric cases, although rare, emphasize the importance of including PVNS in the differential diagnosis of chronic monoarthritis and highlight the need for tailored treatment to preserve growth potential and joint function [8].

Despite advances in imaging, surgical techniques, and adjuvant therapies, the optimal management strategy for PVNS remains controversial. The decision-making process must take into account disease form (localized vs diffuse), anatomic location, patient age and activity level, previous treatments, risk of recurrence, and comorbidities. Furthermore, long-term data on functional outcomes and quality of life are scarce, and no standardized treatment algorithm currently exists.

The aim of this narrative review is to critically evaluate the current evidence regarding diagnosis and treatment options for PVNS.

## 2. Materials and Methods

### 2.1. Search Strategy

A comprehensive narrative review of the literature indexed in PubMed, MEDLINE, and Scopus databases, using search terms: “pigmented”, “villonodular”, “synovitis”, “knee”, “surgery”, and their medical subject headings (MeSH) terms in any possible combination, using the Boolean operators “AND” and “OR”, was performed from January 2000 to December 2024. The reference lists of relevant studies were screened to identify other studies of interest. Both prospective and retrospective studies were considered to ensure a thorough review of the available evidence.

### 2.2. Inclusion and Exclusion Criteria

To ensure the relevance and quality of the included studies, specific inclusion and exclusion criteria were established. Included in this review are studies reporting demographic features, symptoms, diagnostic settings, treatment, possible complications, and outcomes in patients with PVNS. Only articles written in English with available abstracts were included in the study. Excluded from this review were reports of surgical technique, expert opinions, animal studies, unpublished reports, cadaver or in vitro investigations, book chapters, and abstracts from scientific meetings.

### 2.3. Data Collection

Two authors (G.G.M. and A.D.F.) independently conducted the research by title and abstract. If the articles met the inclusion criteria, the full text was obtained and subsequently reviewed. Any discordance was resolved through consensus with a third author (T.G.). All the selected studies were retrospectively analyzed by three authors (G.G.M., C.C., A.D.F.) who then extracted and entered the data in an Excel worksheet. The collected data included: main author, year of publication, number of patients, gender, age, past medical history, clinical presentation, imaging, treatment, and outcomes. Lastly, the data sheet was reviewed by one author (C.P.) who agreed on the extracted data.

## 3. Results

### 3.1. Demographical Data

Following the screening of 173 titles and abstracts, 84 articles were deemed eligible for full-text assessment. Of these, 66 studies met the inclusion criteria and were included in the systematic review. In total, 120 patients diagnosed with PVNS of the knee were analyzed. The mean age of the patients was 25.7 years (range: 1–74). Among them, 20 (16.7%) were pediatric (<18 years) and 3 (2.5%) were elderly (>65 years). The cohort comprised 50 males (41.67%) and 70 females (58.33%). Demographic and clinical characteristics are summarized in Table S1.

### 3.2. Presentation Symptoms and Underlying Conditions

The clinical manifestations of PVNS and related synovial proliferative disorders exhibit notable heterogeneity. Despite this variability, all the patients consistently presented with knee pain and joint swelling, and the majority with mechanical symptoms, including locking, instability, and restricted range of motion. A substantial subset reported chronic or progressively worsening symptoms, with symptom duration ranging from several weeks to multiple years prior to definitive diagnosis. Misdiagnosis in the early stages was not uncommon, with two cases initially at-tributed to juvenile idiopathic arthritis and one case to Familial Mediterranean Fever, thereby contributing to delays in appropriate management [8,24].

Palpable soft tissue masses, typically located in the anterolateral, infrapatellar, or popliteal regions, were frequently associated with localized tenderness and impaired joint function. Mechanical limitations, such as extension block, flexion restriction, and recurrent effusions, were commonly observed and often led to decreased mobility. In more severe presentations, muscle atrophy and alterations in gait mechanics were also noted. While some cases followed a post-traumatic onset, the majority demonstrated an insidious course without clear trauma.

### 3.3. Diagnosis

Diagnostic strategies varied across studies; however, plain radiography (RX) and magnetic resonance imaging (MRI) were commonly employed as standard modalities. Thirty patients (25%) underwent RX as the initial diagnostic test, while 73 (60.83%) underwent MRI. Praino et al. employed arthrocentesis ultrasonography (US) combined with Power-Doppler MRI in three cases [17]. Zhao et al., Kobak et al., and Tatari et al. utilized US in conjunction with RX and MRI [18,25,26]. Liu X et al. [27] reported the use of F-FDG PET/CT (fluorodeoxyglucose positron emission tomography/computed tomography). Bouguennec et al. [28] used CT arthrography as an adjunct diagnostic tool, and Yotsumoto et al. [29] employed angiography. Ares-Rodriguez et al. incorporated electromyography (EMG) in their assessment [30]. In six studies by Kim D.E. et al., Bunting D. et al., Uslu M. et al., Edwards M.R. et al., Dunstan E. et al., and Bojanic et al. [31,32,33,34,35,36], diagnostic methods were not specified, accounting for a total of 12 patients. The definitive diagnosis is confirmed by histopathological analysis.

### 3.4. Treatment Options

The management and treatment of PVNS can involve a combination of preventive measures, intraoperative strategies, and postoperative interventions to mitigate the risk of recurrence. Arthroscopic synovectomy alone was performed in 75 patients (62.5%), and 14 patients (11.67%) underwent arthrotomic synovectomy. A combination of arthroscopic and arthrotomic synovectomy was described in 21 patients (17.5%). Patients treated with a combined approach presented with both intra-articular and extra-articular manifestations of PVNS [2,16,18,22,23,26,31,33,35,37,38,39,40,41,42,43,44]. Klammer G. et al. reported the case of a patient with bilateral diffuse PVNS of the knee, who underwent arthroscopic synovectomy followed by postoperative radiosynoviorthesis on the right knee, and combined arthroscopic and open synovectomy on the left knee [2]. Farthing C. et al. reported a case involving a patient treated with a modified Lemaire extra-articular stabilization [45]. Lalam R.K. et al. presented a series of three patients who underwent radiofrequency thermal ablation [46]. Intra-articular injections of infliximab were administered to three patients at baseline and again after one month under ultrasound guidance, followed by arthroscopic synovectomy five months later (Praino et al.) [17]. One patient was treated with four doses of intra-articular adalimumab (Kobak S. et al.) [25]. Oguz H. et al. administered 15 sessions of intra-articular ozone injections following arthroscopic synovectomy [19]. Gao M. et al. employed an iliac crest bone graft to address a patellar defect after combined arthroscopic and open synovectomy, with postoperative radiotherapy [23]. Chung B.J. et al. reported a case of total knee arthroplasty (TKA) revision due to tibial component loosening in a single patient [47]. Oni J.K. et al. described the first documented case of diffuse PVNS developing within two years after TKA in a patient without pre-existing PVNS [4]. Of note is the study by Kroot E.J. et al., which reported a case treated with arthroscopic excision and open surgical synovectomy, followed by two intra-articular injections of 90Y and a 54-week course of anti-TNFα monoclonal antibody therapy (Infliximab) [40]. Antibiotic therapy was administered in one case (Bouali H. et al.) [41]. Matar H.E. et al. had not reported the type of treatment [48].

### 3.5. Outcomes

The clinical outcomes were reported in 95 cases (79.17%). 85 patients (70.83%) achieved complete recovery, with resolution of symptoms and no recurrence throughout follow-up. Fang Y. et al. and Duan Y. et al. reported two cases of disease recurrence, both managed with arthroscopic synovectomy followed by postoperative radiotherapy [37,49]. Meftah A. et al. described a patient who developed persistent hemarthrosis, ultimately requiring knee arthrodesis due to treatment failure [50]. Lalam R.K. et al. presented a case involving a residual lesion successfully treated with two sessions of radiofrequency ablation [46]. Auregan J.C. et al. described two patients with diffuse PVNS who experienced recurrence, both of whom were treated with arthroscopic synovectomy [9]. Klammer G. et al. re-ported a case of recurrence in the left knee, which was managed with arthroscopic dorsoventral synovectomy combined with open popliteal excision and subsequent radiosynoviorthesis using a yttrium-90 colloid [2]. Yamashita H. et al. described a patient who underwent open synovectomy for recurrent disease [16]. Jobe C.M. et al. reported a patient with three episodes of recurrence, each treated with arthroscopic synovectomy [15]. Mukhopadhyay K. et al. reported a complex case of multifocal PVNS with bilateral joint involvement [51]. The patient initially underwent arthroscopic synovectomy; however, bilateral total knee arthroplasty was ultimately required, and at the latest follow-up, regression of the disease was observed. Finally, Bouali H. et al. reported a case of disease recurrence managed with arthroscopic excision [41]. Ten studies, namely Galli M. et al, Karami M. et al., Tatari H. et al., Xue Liu et al., Kim D.E. et al., Farthing C. et al., Matar H.E. et al., Rajani et al., Garner H.W. et al., and Davidson A. et al., had not reported clinical outcomes [8,14,26,27,31,45,48,52,53,54].

## 4. Discussion

PVNS, or TGCT, is a rare, benign but potentially aggressive proliferative disorder of the synovium. It most commonly affects the knee joint, particularly in adults aged 20 to 50 years, with no sex predilection [3,55]. In our review of 66 studies and 120 patients, PVNS predominantly affects young adults (mean age: 25.7 years), with a slight female predominance (58.3%) and a significant representation of pediatric cases (16.7%), in line with previous reports [3,8,9]. DPVNS was more common than LPVNS, with a higher recurrence rate and technical complexity in management [56,57].

Klammer et al. reported a remarkable case of bilateral diffuse PVNS in a pediatric patient, treated successfully with synovectomies and adjuvant radiosynoviorthesis, emphasizing both the disease’s potential aggressiveness and its presence in younger populations [2].

Chouhan et al. documented multifocal PVNS involving both intra-articular and extra-articular compartments, highlighting the com-plex and unpredictable nature of some presentations [16].

Regarding clinical presentation, our review found that nearly all patients reported monoarticular knee pain and swelling, with many also experiencing recurrent joint effusions and progressive limitation in range of motion. Mechanical symptoms such as locking, instability, or extension block were frequently reported, especially in cases involving intra-articular nodules or anterior cruciate ligament proximity [8,14,17,18,24]. These findings are in line with previous literature, which highlights the non-specific nature of PVNS symptoms and the frequent misdiagnosis as meniscal injuries, synovial cysts, or inflammatory arthropathies [1,9,55,58,59,60,61,62,63]. A small number of patients also presented with palpable masses or extra-articular swelling, especially in cases of diffuse or multifocal disease [16,29]. The heterogeneity of clinical presentation may contribute to delayed diagnosis and underlines the need for increased awareness among clinicians when evaluating persistent monoarthritis in young adults or pediatric patients.

Our findings confirm that MRI is the gold standard for diagnosis due to its high sensitivity for hemosiderin detection, consistent with NCCN guidelines [19,64]. However, we also identified several advanced diagnostic modalities, such as 18F-FDG PET/CT [59], angiography [29], CT arthrography [28], and ultrasonography [17,18,25,26], particularly useful in complex or recurrent presentations. These tools may enhance diagnostic accuracy and preoperative planning in selected cases.

Histologically, PVNS is characterized by hyperplastic synovium with hemosiderin-laden macrophages, multinucleated giant cells, and lipid-laden histiocytes [55].

Molecular studies have identified CSF1 overexpression as a key factor in the pathogenesis of PVNS, reinforcing the rationale for targeted therapies [21]. As mentioned in the NCCN Soft Tissue Sarcoma Guidelines, this finding supports the use of CSF1R inhibitors, most notably pexidartinib for symptomatic, unresectable, or relapsing TGCT/PVNS, highlighting the importance of a molecularly driven therapeutic approach [64].

Surgical synovectomy remains the gold standard of treatment [64]. Our review found that arthroscopic synovectomy was performed in 62.5% of cases, while open or combined open-arthroscopic approaches were used in 11.7% and 17.5% of cases, respectively. The latter were mainly employed in diffuse or extra-articular disease [2,16,18,22,23,26,31,33,35,37,38,39,40,41,42,43,44]. This aligns with prior literature reporting lower recurrence with open or combined procedures [60,61,62]. Sharma et al. reported a recurrence rate of 92% after isolated arthroscopy versus 19% following open procedures [61], while Colman et al. suggested that a combined anterior arthroscopic and posterior open approach could reduce recurrence to 9% [62]. According to Auregan et al., arthroscopic synovectomy in diffuse PVNS yielded excel-lent functional outcomes, achieving a 91% success rate at 7-year follow-up when complete resection of the synovium was obtained [9].

Postoperative rehabilitation is critical for preserving joint function. Functional outcomes following synovectomy were generally favorable across the studies reviewed, particularly in cases where complete resection was achieved [16,31,44,63]. Most patients reported significant reduction in pain and joint swelling, as well as improvement in range of motion and daily activity performance [33,44,63]. However, formal evaluation of functional scores, such as the Lysholm or Tegner activity scale, was inconsistently reported across studies, limiting a more robust comparison. Postoperative follow-up protocols were also variable, though routine MRI at one year was commonly used to detect subclinical recurrence. Auregan et al. [9] reported that although all patients had negative MRI findings at 12 months, two experienced clinical relapse at year 2 and year 5, reinforcing the need for long-term radiological monitoring beyond the first postoperative year. The heterogeneity in follow-up duration and reporting across the included studies highlights the need for standardized outcome measures and imaging intervals to optimize long-term surveillance and functional assessment in PVNS patients.

Recurrence, reported in 14–56% of cases, remains the major challenge [60,61,62]. In our cohort, 20.8% of patients experienced recurrence, particularly in diffuse disease or after limited resection. Risk factors included posterior or extra-articular involvement [2,62], prior surgery, and possibly iatrogenic causes. Rare presentations in locations such as Hoffa’s fat pad and patellar tendon sheath may also complicate diagnosis and contribute to recurrence [65,66,67,68]. Do Cho et al. described recurrence after ACL reconstruction with synthetic graft, suggesting a potential link to foreign-body reaction [68].

Adjuvant treatments were reported in a minority of cases but showed promising outcomes. Radiosynoviorthesis with yttrium-90 or osmic acid was used in selected cases, especially when margins were incomplete or recurrence occurred [9,67]. Biologic therapies (e.g., infliximab, adalimumab) were administered intra-articularly in patients with refractory or recurrent disease, with symptomatic and histological improvements [17,25,40]. Ozone therapy was also employed in one case [19]. These therapeutic options, although not uniformly established in guidelines, reflect the evolving landscape of personalized PVNS management.

The introduction of CSF1R inhibitors such as pexidartinib has expanded systemic treatment options. Tap et al. reported significant tumor regression and symptom relief in unresectable PVNS [67], although concerns regarding hepatotoxicity remain. Our review supports the use of these agents in select cases, while highlighting the need for long-term safety data.

Our findings align with the current NCCN guidelines, which recommend MRI as the imaging modality of choice and surgical synovectomy as the standard treatment for both localized and diffuse forms of PVNS. Furthermore, they support the use of CSF1R inhibitors such as pexidartinib in cases of symptomatic, unresectable, or relapsing disease. 

We performed a comprehensive evaluation of both conventional and emerging imaging modalities, including PET/CT, angiography, CT arthrography, and ultrasonography, which are not explicitly detailed in the NCCN guidelines but may enhance diagnostic accuracy in complex or recurrent cases. Additionally, our data highlight the importance of combined surgical approaches (arthroscopic plus open techniques) in reducing recurrence rates in diffuse PVNS, an aspect only briefly touched upon in existing protocols.

We also emphasize the integration of adjuvant and systemic therapies such as radiosynoviorthesis, biologic agents (e.g., infliximab, adalimumab), and ozone therapy in selected patients, especially in cases of recurrence or incomplete resection. These treatment options, while not yet uniformly recommended in the guidelines, reflect the evolving landscape of personalized PVNS management.

Finally, we have shown the rare and underrepresented clinical scenarios, such as pediatric PVNS, cases following total knee arthroplasty, and multifocal or extra-articular forms. By systematically including these variants, we aim to broaden the clinical awareness and guide tailored management strategies in less typical presentations, which are often overlooked in standard guideline frameworks.

## 5. Conclusions

Pigmented villonodular synovitis (PVNS) of the knee is a rare but potentially aggressive condition that necessitates a multidisciplinary approach. Accurate and timely diagnosis, comprehensive preoperative imaging, and complete surgical excision are essential to minimize the risk of recurrence and preserve joint function. In cases of diffuse or recurrent disease, a combined open-arthroscopic synovectomy should be considered to im-prove surgical access and outcomes. Furthermore, in patients with particularly high risk of relapse or incomplete resection, adjunctive modalities such as radiosynoviorthesis or systemic therapies targeting the CSF1–CSF1R axis may be valuable options within an individualized treatment plan. This narrative review is subject to several limitations, primarily due to the nature of the included studies. Most of the literature analyzed consists of case reports or small case series, which inherently carry a low level of evidence and a high degree of heterogeneity. As such, the generalizability of the findings remains limited, and definitive conclusions regarding best practices must be interpreted with caution.

Future research should focus on high-quality, multicenter, prospective studies with standardized diagnostic and treatment protocols. Comparative analyses of surgical approaches, long-term follow-up data, and the evaluation of adjuvant and systemic therapies will be crucial to establish evidence-based management algorithms and optimize patient outcomes.

## Data Availability

No new data were created or analyzed in this study. Data sharing is not applicable to this article.

## Supplementary Materials

The following supporting information can be downloaded at: https://www.mdpi.com/article/10.3390/jcm14165857/s1, Table S1: Demographic data, clinical presentation, diagnostic tools and treatments of all the patients included in the study [68,69,70,71,72,73,74,75,76,77,78,79,80,81,82,83,84,85,86,87,88,89,90].

## Abbreviations

The following abbreviations are used in this manuscript:

PVNS	Pigmented villonodular synovitis
TGCT	Tenosynovial giant cell tumor
LPVNS	Localized PVNS
DPVNS	Diffuse PVNS
CSF1	Colony-Stimulating Factor 1 (CSF1)
CSF1R	Colony-Stimulating Factor 1 inhibitor
