# Supplementary material for: Diagnosis and Treatment Options in Pigmented Villonodular Synovitis of the Knee: A Narrative Review
Source: J Clin Med. 2025 Aug 19;14(16):5857. doi: 10.3390/jcm14165857 (PMC12387440; doi:10.3390/jcm14165857)
Supplement: Supplementary file 1 [file jcm-14-05857-s001.zip › jcm-3747558-supplementary.pdf]

Table S1. Demographic data, clinical presentation, diagnostic tools and treatments of all the patients included in the study.

Author	Year	No. Patients	Age	Gender	Past medical history	Clinical Presentation	Imaging	Treatment	Outcomes
Degirmenci E et al. [69]	2020	1	27	M	Trauma 1y prior	Palpable cystic structure at the lateral border of the patellar tendon, pain, restricted ROM	RX, MRI	Open synovectomy	Full recovery
Liu, X et al. [27]	2021	1	37	F	-	Pain, restricted ROM in flexion	MRI,F-FDG PET/ CT	Arthroscopic synovectomy	-
Xu D et al. [70]	2021	1	58	F	Hyperuricemia, trauma 20y prior	Tender palpable soft tissue mass in the the left knee, pain, swelling, restricted ROM	MRI	Open synovectomy	Full recovery
Oguz H et al. [19]	2020	1	30	F	Arthroscopic synovectomy 10y earlier for PVNS	Pain, swelling, limited ROM in flexion	RX, MRI	Arthroscopic synovectomy + 15 sessions of intrarticular ozone injections	Full recovery
Fang Y et al. [37]	2020	1	21	F	-	Progressive pain	MRI	Arthroscopic synovectomy (anterior approach) and open synovectomy (posterior approach)	Recurrence: Athroscopic synovectomy 20 months later, followed by radiotherapy 6 weeks post-surgery
Do Cho S et al. [68]	2020	1	52	F	ACLR 21y earlier using an active bioprosthetic composite artificial ligament	Pain, swelling, instability	MRI	Arthroscopic synoviectomy	Full recovery
Turkucar S et al. [24]	2019	4	14	M	-	Pain and swelling, misdiagnosed as juvenile idiopathic arthritis (JIA)	MRI	Artrhoscopic synovectomy	Full recovery
			1	F	-	Swelling for 3 months after minor trauma; suspected as oligoarticular JIA	MRI	Open synovectomy	Full recovery
			11	F	Compound heterozygote mutations A744S/R202Q in MEFV gene	Pain and swelling accompanied by abdominal pain; suspected as Familial Mediterranean Fever (FMF).	MRI	Open synovectomy	Full recovery

			5	F	Family history of FMF	Pain and swelling following minor trauma, suspected FMF	MRI	Artrhoscopic synovectomy	Full recovery
Matar HE et al.[48]	2019	1	14	M	-	Pain, swelling	MRI	-	-
Kim DE et al. [31]	2018	10; 6	32; 32	F; M	Diffuse extra-articular PVNS/TGCT	Pain, swelling, and limited ROM	MRI	Arthroscopic synovectomy (8 patients); open posterior synovectomy (1 patient); combined arthroscopic and open approach (7 patients)	-
Farthing C et al. [45]	2018	1	21	M	Chronic PVNS, several arthroscopic synovectomies, partial meniscectomies, and meniscus repairs	Pain, swelling, pivot shift grade 3	MRI	Modified Lemaire extra-articular stabilization	-
Karami M et al. [8]	2018	1	3	M	Previous treatment with growth hormone for 1y, misdiagnosed as Juvenile Idiopathic Arthritis (JIA)	Pain, swelling, and patellar dislocations	RX, MRI	Open synovectomy	-
Duan Y et al. [49]	2018	1	27	F	-	Pain, swelling, and limited ROM	Joint puncture, CT-biopsy, MRI	Open synovectomy	Recurrence after 2y : arthroscopic sinovectomy + RT (15 sessions, 30 gray)
Rajani R et al. [52]	2018	1	42	F	ACL reconstruction	Pain, instability	RX,MRI	Open synovectomy	-
Falster C et al. [71]	2017	1	24	F	-	Pain, reduced ROM, Apley +	MRI	Arthroscopic synovectomy	No recurrence after 1y folllow up
Gao M et al.[23]	2017	1	37	F	-	Pain, reduced ROM, mass in popliteal fossa	RX, MRI	Arthroscopic total synovectomy, open resection of the popliteal fossa mass, and iliac crest bone graft for patellar defect + 50% zinc chloride applied to the patellar cavity as an adjuvant treatment + RT (15 sessions total 20 gray)	No recurrence after 1y follow up
Keyhani S et al. [22]	2025	1	5	F	-	Gradual, progressive pain and swelling	RX, MRI	Open + arthroscopic synovectomy	No recurrence at 18 months follow up
Meftah A et al. [50]	2016	1	28	M	Diabete Mellitus 1	Pain and intermittent swelling in both knees, restricted ROM	RX, MRI	Open synovectomy	Persistent hemarthrosis post-synovectomy; arthrodesis

Camp CL et al. [72]	2016	1	64	F	TKA 9y prior, LUPUS, anticoagulant for Antiphospholipid antibody syndrome	Patella fracture, flexion instability	RX,	Revision TKA + open synoviectomy	Full recovery at 18 months follow up
Lalam RK et al. [46]	2015	3	22	F	2 unknown arthroscopic procedures	Pain, swelling	MRI	CT biopsy; Radiofrequency Thermo-Ablation (RF Ablation)	Residual lesion detected at 6-month follow-up treated with 2 RF ablation
			27	F	-	Pain, swelling	MRI	CT biopsy; Radiofrequency Thermo-Ablation (RF Ablation)	No recurrence detected at 6 years follow up
			29	M	-	Pain, swelling	MRI	CT biopsy; Radiofrequency Thermo-Ablation (RF Ablation)	No recurrence detected at 6 years follow up
Praino E et al. [17]	2015	3	47	F	Open synovectomy + methrotrexate + prednisolone	6 months recurrence of PVNS	Arthrocentesis Ultrasonography (US) with Power-Doppler MRI	Intrarticular Infliximab injections time 0, 1 month, with US + Synovectomy at 5 months	No recurrence at 12 months
			51	F	Open synovectomy + methrotrexate + prednisolone	6 months recurrence of PVNS	Arthrocentesis Ultrasonography (US) with Power-Doppler MRI	Intrarticular Infliximab injections time 0, 1 month, with US + Synovectomy at 5 months	No recurrence at 12 months
			57	M	Open synovectomy + methrotrexate + prednisolone	9 months recurrence of PVNS	Arthrocentesis Ultrasonography (US) with Power-Doppler MRI	Intrarticular Infliximab injections time 0, 1 month, with US	No recurrence at 12 months
True VL et al. [73]	2015	1	8	F	Chediak-Higashi Syndrome , Turner Mosaicism	Pain, swelling, intetrmittent fever, anemia	MRI, Biopsy	Prednisole + Cyclosporine + open synovectomy and debridment	Estimated 80% resection but no recurrence after 10y
Zhao X et al. [18]	2014	1	57	M	Rheumatoid Arthritis	Pain and swelling in the right knee	Musculoskeletal Ultrasound, MRI, Biopsy via arthroscopy	Arthroscopic synovectomy + mini open + methotrexate (MTX) 10 mg/week for RA	Full recovery at 1y follow up
Bouguennec N et al. [28]	2014	1	31	M	-	Pain, swelling, Apley+	RX, MRI, CT arthrography	Arthroscopic synovectomy + Meniscal suture	No recurrence at 2y follow up
Auregàn J.C et al. [9]	2013	13	41	M	-	Pain, swelling, mechanical symptoms, and limited ROM	MRI	Arthroscopic synovectomy	No recurrence at 7 years
		10	41	F	-	Pain, swelling, mechanical symptoms, and limited ROM	MRI	Arthroscopic synovectomy + disease with residual lesions using Osmic acid (until 2007) or	2 patients with recurrences:

								Triamcinolone hexacetonide thereafter	arthroscopic synovectomy
Klammer G et al. [2]	2013	1	16	F	Family history of thrombophilia, mild von Willebrand disease, heterozygous prothrombin gene mutation	Intermittent anterior left knee pain during sports (gymnastics, horse riding), without previous trauma, LATER, pain also developed in the right knee	MRI	LEFT KNEE Arthroscopic synovectomy (intrarticular part) + Open excision of extrarticular popliteal mass RIGHT KNEE Arthroscopic synovectomy + radiosynoviorthesis postop	LEFT KNEE Recurrence 3.5y after surgery: arthroscopic synovectomy and open popliteal excision with radiosynoviorthesis an yttrium-90 colloid 20 months later
Yamashita H et al. [16]	2012	1	14	F	-	Pain, two tender palpable masses: one anteromedial and one posteromedial, restricted ROM	MRI	Intraarticular mass: Arthroscopy Extra-articular mass: Open surgery	Recurrence at 2y follow up: open synovectomy
Galli M et al. [14]	2012	1	12	F	-	Pain, pain after 90° of flexion	RX, MRI	Arthroscopic synovectomy	-
Jobe CM et al. [15]	2011	1	32	F	-	Pain	MRI	Arthroscopic synovectomy (2 times)	Recurrence (3 times): Arthroscopic synovectomy with removal of neosynovium, periarticular fat, and areolar tissue.
Oni JK et al. [4]	2011	1	73	M	TKA 18y prior	Pain, swelling	Blood test, RX, arthocenteisis, arthroscopy	Arthroscopic synovectomy	No recurrence at 6 months follow up
Chung BJ et al. [47]	2011	1	74	F	TKA 5y prior	Pain, swelling	RX	Revision TKA (tibial component loosening)	No recurrence after 1y
Boussaadani Soubai R et al. [74]	2011	1	20	F	-	Progressively intermittent pain and swelling of both knees	RX, MRI	Open synovectomy + lateral release+ vastus medialis obliquus advancement	Full recovery
Kobak S et al.[25]	2011	1	54	F	-	Pain, swelling and limited ROM	RX, MRI, US	Four doses of intraarticular adalimumab	Full recovery at the 6th month follow-up visit
Lee KH et al. [75]	2010	1	43	M	Left knee arthroscopy 15y prior	Painful intermittent knee locking	MRI	Artrhoscopic excision	Full recovery
Chae DJ et al. [76]	2009	1	23	M	4 knee aspirations	Knee dull pain and swelling, aggravated by any athletic activity	RX, MRI	Artrhoscopic excision	Full recovery
Hegedus EJ et al. [77]	2008	1	46	F	Hypothiroidsm, RT for thyroid cancer	Self-limiting, intermittent left knee pain, dysfunction,	MRI	Artrhoscopic synovectomy	Full recovery

						severe swelling and locking			
Garner HW et al. [53]	2008	1	51	M	-	Pain, swelling	RX, MRI	Artrhoscopic excision	-
Yotsumoto T et al. [29]	2008	1	26	M	-	Locking symptoms	MRI, Angiography	Artrhoscopic excision	Any recurrence of pain or locking symptoms after 2 years
Ares-Rodriguez et al. [30]	2007	1	39	F	Arthroscopy; anterior arthrotomy with subtotal synovectomy and lateral release (Ficat); posterior arthrotomy	Pain at rest and when walking; lumpy knee	RX, MRI, EMG	Synoviorthesis with Yttrium 90 (90Y); after 10 months, posterior arthrotomy with synovectomy	Full recovery
Bunting D et al. [32]	2007	1	71	F	bTKA	Spontaneous onset of painful episodes in right knee and associated swelling 15 months after surgery	-	Artrhoscopic resection 22 months after surgery	Full recovery
Kanagawa H et al. [78]	2007	1	31	M	Several aspirations of brownish joint fluid	Intermittent pain and swelling	MRI	Arthroscopy of the right knee one month after a twisting injury	Completely asymptomatic with full range of motion of the knee
Yoo JH et al. [38]	2007	1	35	M	-	Dull pain	RX, MRI	Arthroscopic examination and excisional biopsy	No evidence of recurrence at one year postoperative
Kakarala G et al. [79]	2007	1	50	F	-	Anteromedial pain, occasional popping without frank locking or giving way	RX, MRI	Arthroscopic excision	Asymptomatic at the 18-month follow-up
Brenner JS et al. [39]	2007	1	17	F	3 knee aspirations of serosanguineous fluid	Painless chronic knee effusion, insidious onset to the swelling	RX, MRI	Arthroscopic biospy and synovectomy; after 8 months, open anterior and posterior synovectomy and radical soft tissue resection of the disease within the extracapsular popliteal fossa	Full recovery

Neubauer P et al. [80]	2007	4	8	M	-	Swelling	MRI	Arthroscopic synovectomy x3	No evidence of recurrence after 5 years
			11	F	-	Sudden painful swelling	RX, MRI	Arthroscopic synovectomy	No recurrence one year post-op
			11	F	-	Swollen and tender knee	MRI	Arthroscopic synovectomy	Full recovery at 2 years post-op
			15	M	-	Increasing knee pain and swelling following a car accident	MRI	Arthrotomic synovectomy	No evidence of recurrence after 3 years
Uslu M et al. [33]	2006	1	19	F	Pregnant at the 10th week; 2 knee aspirations	Severe pain and swelling with difficulty in walking and flexing the knee	-	Diagnostic arthroscopy, arthrotomic excision	Asymptomatic at 2 year follow-up
Mukhopadhyay K et al. [51]	2006	1	5	F	Knees aspirations	Pain and swelling in her knees	MRI	Synovectomy x2, intra-articular corticosteroids	bTKA and multiple joints PVNS; signs of regression of disease in her last out-patient review
Stubbs AJ et al. [81]	2005	1	26	F	-	Atraumatic pain with increased swelling and tenderness in the popliteal fossa	RX, MRI	Arthroscopic synovectomy	Full recovery
Kim RS et al. [82]	2005	2	40	F	-	Palpable lump in the infrapatellar area of the right knee and gradually increasing discomfort according to knee flexion and eliciting pain with full flexion	MRI	Arthroscopic excision	No evidence of recurrence at one year postoperative
			12	M	Blunt trauma 6 months previously	Aggravated pain when bending the left knee, tenderness on the infrapatellar area	MRI	Arthroscopic excision	No recurrence during a 14-month follow-up period
Tavangar SM et al. [83]	2005	1	5	F	-	Increasing knees pain not respond to analgesic	RX, MRI	Arthroscopic excision	Multifocal PVNS
Kroot EJ et al. [40]	2004	1	22	M	-	-	MRI	Arthroscopic excision; open surgical synovectomy; 2 intra-	Full recovery without side effects

								articular injections of 90Y; anti-TNFa monoclonal antibody (infliximab) treatment for 54 weeks	
Hantes ME et al. [84]	2004	1	42	F	-	Knee pain, repeated episodes of locking and inability to make a full flexion of the knee	MRI	Arthroscopic excision and thermal debridement of the synovial attachment site	Asymptomatic and no signs of recurrence 1y post-op
Bouali H et al. [41]	2004	1	26	M	-	Painless swelling and tightness	MRI	ATB; cortico-steroid injections; arthroscopy and surgical synovectomy	Recurrence one year after surgical resection; arthroscopic synovectomy; full functional capacity and symptom-free
Edwards MR et al. [34]	2004	1	19	F	Pregnant; knee injury with no bony involvement 8y prior	Left monoarticular anterior knee pain and clicking; movement precipitated the clicking; lateral tracking of patella to a mild extent	-	No improvement of symptoms with the physiotherapy; arthroscopy excision	At 6-month follow-up complete resolution of symptoms
Lu KH et al. [42]	2004	1	32	F	Open excisional biopsy for right knee pain with a popliteal cyst 9y prior	Progressively enlarging nodule in the antero-lateral arthroscopic scar of the right knee with some limitation of flexion	MRI	Arthroscopic examination through the anterolateral portal and an anterior open synovectomy simultaneously for diffuse PVNS of the right knee	2 small residual extra-articular extension lesions due to portal drain contamination; tumor excision; full recovery
Sansone V et al. [43]	2004	1	41	F	Removal of a popliteal cyst using an open posterior approach	Symptomatic enlarging mass in the right popliteal fossa	RX, MRI	Simultaneous arthroscopy followed by an open resection of the popliteal cyst	Rapid return to full range of motion and physical activity, symptom free
Saulsbury FT et al. [85]	2004	1	9	F	Pauciarticular juvenile rheumatoid arthritis; right knee arthrocentesis	Swollen knee	MRI	Arthroscopic synovectomy	Very small residual effusion of the right knee at 1y postoperative; fully functional and normally active
Roach R et al. [44]	2003	1	30	M	-	Locking and recurrent swelling knee	RX, MRI	Arthroscopy and a mini-arthrotomy excision	Immediate resolution of symptoms and rapid return to sport

Kim RS et al. [86]	2003	1	28	M	Previous left knee trauma; partial medial meniscectomy of the same knee	Gradually increasing discomfort and aggravating knee pain on squatting	RX, MRI	Artrhoscopic excision	No evidence of recurrence at 2y follow up
Dunstan E et al. [35]	2002	2	26	M	-	Intermittent knee locking	RX, MRI	Artrhoscopic excision	Full recovery
			31	M	-	Lump in his right knee		Arthroscopy and a mini-arthrotomy excision	Full recovery
Parikh SN et al. [87]	2002	1	46	M	-	Vague anterolateral knee pain not associated with activity; intermittent, dull ache that unrelated to knee position	MRI	Diagnostic arthroscopy; arthroscopic excision of the lesion, aggressive debridement of the synovial attachment site	Return to full activity by 4 weeks
Bojanic I et al. [36]	2001	3	60	M	-	Swollen and painful knee, mechanical block to the full extension (range of motion 20°–90°), marked atrophy of the left quadriceps muscle	-	Arthroscopic resection	At 10-month follow-up no signs of clinical recurrence
			41	F	Skiing trauma 5y previously	Pain when bending the left knee, with occasional jump-overs and movability of the loose body in the joint	RX	Arthroscopic excision	Completely asymptomatic at 1y follow-up
			22	F	Injury while playing badminton one year previously	Pain and episodes of swelling; mechanical block to full extension (range of motion 15°–120°)	-	Arthroscopic excision	Symptom free at 8-month follow-up
Aşik M et al. [88]	2001	4	36	M	-	Initial symptoms of swelling and restriction of flexion	MRI	Arthroscopic excision	Totally free of symptoms after 18 months
			19	F	-	Symptoms of anterior knee pain and a clicking sensation during extension; palpable	MRI	Diagnostic arthroscopy and excision	Return to daily activities at the end of the first postoperative week and, after 2y, totally free of symptoms

						mass medial to the patella			with no finding of recurrence
			59	F	-	Pain in the medial part of knee, worse at night	MRI	Arthroscopic examination and excision	After a follow-up period of 2y, minimal pain during and after daily activities, no sign of recurrence
			26	F	-	Pain in the lateral and ventral parts of her knee, temporary episodes of effusion, and locking sensation	MRI	Arthroscopic examination and excision	Complete recovery of symptoms with no apparent recurrence after 2y
Davidson A et al. [54]	2001	1	31	M	Traumatic dislocation of the patella	Disabling pain, persistent intermittent effusions, instability, and giving way; irritating herniating loose body at the joint line lateral to the patella	Rx	Arthroscopic excision	-
Bartlett MJ et al. [89]	2000	1	31	F	-	Recurrent lateral dislocation of left patella and anterior knee pain	MRI	Arthroscopic resection	Symptom free at 1y post-op
Tatari H et al.[26]	2000	1	60	M	-	Increasing pain and swelling in the posterior aspect of knee	MRI, US	Open excisional biopsy; arthroscopic debridement	Full recovery
Dale K et al. [90]	2000	1	25	F	Several times drainage and instillation of steroid in the knee	Swelling and discomfort of the knee	Rx, MRI	Arthroscopic biopsy with diagnosis of synovitis; arthroscopic synovectomy after 1y	Full recovery
